# How does firms' broadband adoption affect regional TFP in Italy?

**DOI:** 10.1007/s40888-022-00269-5

**Published:** 2022-06-13

**Authors:** Massimo Giannini, Barbara Martini, Cristiana Fiorelli

**Affiliations:** Department of Business Engineering, University of Rome Tor Vergata, Rome, Italy

**Keywords:** Broadband, TFP, Firms behaviour, Spatial panel, O33, O38, C23, D22

## Abstract

In the literature, the positive effect of ICT on labour productivity and, in general, economic growth is vast and well consolidated. This paper wants to go beyond the general term of ICT and look inside the "black box." In particular, broadband adoption among Italian firms is critical for productivity. Hence, we focus on broadband adoption and internet facilities and how they affect the firms' total factor productivity in the Italian business sector firms at NUTS2 over the period 2003–2018. Italy is indeed still characterized by a robust North–South divide. Our question is: can we exploit the digital advantage for filling the productivity gap? To answer, we are going to use a classical two-stage approach. In the first one, the TFP is filtered out using both a semi-parametric approach and a parametric one (spatial ML). The second step investigates its determinants, using broadband firms' adoption as a covariate in an ECM augmented by spatial spillovers, controlling for competitivity, internationalization, and human capital. Our results show a positive relationship between TFP and broadband adoption (cointegration), including regional spillovers; this positive effect spreads to GVA. Moreover, our results show that digitalization makes Southern regions more resilient to external shocks.

## Introduction

The Next Generation EU plan is the ambitious social and economic strategy that should drive the EU out of the Covid crisis. In Italy, the Government has launched the National Plan for Recovery and Resilience, representing the implementation of the EU plan. The Italian plan is composed of six pillars. In particular, the pillar related to digitalization, innovation, and competitivity (Mission 1) plays a prominent role in the recovery phase, including the rebalance of the North–South divide in Italy. The financial resources for such mission amount to 21% of the entire national plan budget (the latter is about 211 billion of Euro plus another 100 coming from the National Budget). In particular, the investments for the business sector's digitalization and innovation amount to 38 billion euros. In mission 1, a specific point is devoted to developing broadband and 5G in the SMEs (4.2 billion). Such investments should speed up the Italian Strategy for the ultra-broadband network launched in 2015. It addressed to cover, within 2020, 85% of the population with a speed of at least 100 Mbps (NGA-VHCN -Very High-Capacity Networks) and the full coverage of the population with at least 30 Mbps.

As said, the broadband investments lie under the mission of increasing the competitiveness of the business sector; nonetheless, the National Plan does not underline how broadband coverage and adoption will be transferred in a higher competitivity. Presumably, the policymaker refers to the economic literature related to the positive effect of ICT investments on firms' productivity, notably labour. This literature is vast and well consolidated. As Italy is concerned, Iammarino and Jona-Lasinio (2013) analyse the relationship between ICT and labour productivity in the Italian regions in 2001–2005. The authors find: "a strongly positive relationship between ICT production and regional labour productivity growth, at the same time suggesting a complementary relationship between ICT production and diffusion in explaining interregional differences in productivity performances" (page 218). Hall et al. (2013) investigate the effect of investments in R&D and ICT on innovation and productivity of Italian firms over the period 1995–2006. They find that "ICT and R&D contribute to productivity both directly and indirectly through the innovation equation, but they are neither complements nor substitutes. However, individually they each appear to have large impacts on productivity, suggesting some underinvestment in these activities by Italian firms." (page 318).

From a theoretical point of view, human capital and technology have been crucial drivers of economic growth since the pioneering contributions by Lucas (1988), Romer (1990), and Aghion and Howitt (1992). In the last twenty years, the importance of ICT, with particular attention to the digital revolution, is even more evident; in particular, high-speed internet via broadband infrastructure may affect the innovative capacities of the economy through the development of new products, processes, and business models to promote growth. Moreover, the availability of more extensive and sometimes accessible information knowledge involves external effects that facilitate the adoption and sharing of new technologies, fostering economic growth (see the pioneering contribution by Nelson and Phelps, 1966).

From the policymaker viewpoint, fostering the digital economy has been on the European agenda since the Industry 4.0 action plan, until the "Shaping Europe's Digital Feature" in February 2020. In the last few years, many efforts have been devoted to providing data and measures of digitalization. The Digital Economic and Society Index (DESI) is an example. Following the vast literature on ICT and human capital, the index covers five dimensions of the digital revolution, summarized in Table 1 of the DESI 2020 report:

1. Connectivity—Fixed broadband take-up, fixed broadband coverage, mobile broadband, and broadband prices.
2. Human Capital—Internet user skills and advanced skills.
3. Use of Internet—Citizens' use of internet services and online transactions.
4. Integration of digital technology—Business digitization and e-commerce
5. Digital public services—e-Government.

The 2020 DESI for Italy raises several questions about points 1–5. First, looking at the general index, Italy performs poorly. It is 25 out of the 28 EU members. However, this poor performance is mainly due to the low human capital: "Compared to the EU average, Italy records shallow levels of basic and advanced digital skills. ICT specialists and ICT graduates are also well below the EU average. These gaps in digital skills reflect the poor use of online services, including digital public services. Similarly, Italian enterprises lag in the use of technologies such as cloud and big data, as well as in the uptake of e-commerce" page (3). About the connectivity sub-index, Italy ranks 17th among EU countries (we will come back on the point in the following section).

This paper will focus on points 1 and 2 and their relationship with Italian firms' Total Factor Productivity (TFP). We want to investigate how broadband adoption among Italian firms affects their productivity. Answering this question is crucial to assess whether the Recovery Plan will effectively support firms' competitiveness, hence growth. Theoretically, the widespread of broadband fosters the sharing and availability of information and data across multiple locations; moreover, it opens to a new opportunity for firms in e-commerce, information availability, market perspectives, etc. Beyond the reduction of costs of existing business processes, high-speed internet enables new business and firm-cooperation models that rely on the spatial exchange of local information, which fosters competition and innovation processes. On the demand side, broadband internet may increase market transparency and thus additionally intensify competition. However, an empirical answer for the Italian case has to be provided.

When combined with information technologies, the broadband infrastructure can also affect firm productivity and economic growth in additional ways. The development of information technologies fundamentally changed and improved information processing, resulting in significant productivity growth of IT-using firms (Stiroh, 2002). The recent literature on productivity effects of information technologies (IT) also recognizes that these effects depend on the use of IT and the presence of complementary inputs such as skilled labour (Autor et al., 2003) or organizational structure and practices (Bresnahan et al., 2002; Bloom & Van Reenen, 2007; Bloom et al., 2010; Cappelli et al., 2010).

More recently, Gal et al. (2019) provided a comprehensive analysis of the effects of the digital economy on industry productivity in European countries: "Our findings support the idea that the adoption of digital technologies is generally associated with substantially higher firm-level productivity. These results hold for various technologies (high-speed broadband access, simple and complex cloud computing, CRM, and ERP software). Moreover, the association between the adoption of digital technologies and productivity is also more reliable for firms that are already highly productive, hence likely to benefit from complementary organizational and technical skills" (page 31).

Nonetheless, there are no specific empirical contributions on the effects of broadband adoption on the TFP in Italy; this is the novelty of our contribution. We address the question from a regional perspective for two reasons. Firstly, our primary goal is to assess the effect of broadband adoption in Italian firms in fostering regional development. Broadband coverage and adoption lie in the EU strategic framework for regional development and social cohesion and is an instrument for such a policy, even before the Next Generation EU. Italy is part of this framework; both the Italian Digital Agenda plan and the Italian Strategy for Ultra-Broadband are examples. The Minister for Infrastructures and Transports (MIT), the one for Economic Development (MISE) and the one for the Technological Innovation and Digital Transition (MITD) manage the targets that Italy has established with the EU. Secondly, reliable time series on firms' network adoption does not exist at the firm level. The official data comes from the Italian National Institute for Statistics (Istat—which provides Eurostat data to the EU commission for monitoring the implementation of the network) but only at a regional level (Italy NUTS 2). Moreover, the regional dimension allows us to analyse the possibility of spatial spillovers generated by widespread internet firms' adoption and higher human capital; more connected territories represent another channel through which new ideas, processes, and organizations spill over into the firms.

Our results confirm a positive relationship between ICT facilities and TFP. Under this point of view, it seems evident that policies fostering broadband, pc, and internet adoption in the Italian firms increase their productivity. Such a result holds both for Northern and Southern firms, although differences remain. Especially in the South, the increase in broadband usage has contrasted with the fall of TFP after the 2008 financial crisis. Since 2014, regions have shown a gross value-added recovery trend (GVA). For the Northern areas, this recovery was mainly linked to improved employment levels, and a less extent, in the TFP (except Far North–East). By contrast, in the Southern regions, the expansion of TFP has supported the recovery of the GVA. This is because the Southern companies have gained more from digital infrastructures.

In general, broadband adoption sustains TFP, and it positively impacts GVA, tempering the negative impact of a recession. Moreover, the spatial estimate shows that positive spatial spillovers among neighbouring regions are at work. Under this point of view, sustaining the digital economy could be promising for the post-Covid age.

The paper is organized as follows. In the next section, we present some stylized facts about firms' broadband adoption in Italy. Sections 3 and 4 estimate the TFP, while Sect. 5 investigates the relationship between TFP and ICT component. Discussion of main findings and policy implications are in Sect. 6. Section 7 concludes.

## Some stylized facts

Istat, the National Institute for Statistics, collects data on broadband adoption in private firms with more than ten employed since 2003; although this firm size covers only 10% of the total, they represent more than 50% of GDP and employment. However, the definition of broadband itself is not fully clear; in Italy,^1^ it represents a fixed network with at least two megabits per second (MBPS). The Telecommunication Standardization Sector (International Telecommunication Union), with the I.113 (06/97) standard, defines broadband as a transmission higher than the primary rate ISDN, i.e., 1.5 (in the USA) and 2 MBPS in Europe. Nonetheless, for the European Commission, the broadband involves at least 30 MBPS in downloading—NGA (Next Generation Access or ultra-broadband).

The European Digital Agenda defines three levels of broadband speeds: 2, 30, and 100 MBPS. On the third of March 2015, the Italian Government signed the "Strategia Italiana per la banda ultralarga" (Italian Strategy for the ultra-broadband network) addressed to cover, within 2020, 85% of the population with a speed of at least 100 Mbps (NGA-VHCN -Very High-Capacity Networks) and the full coverage of the population with at least 30 Mbps. The European Cohesion Fund finances the Strategy with 3,5 billion and a further 1,8 billion from national and regional development funds. Additional funds will come from the National Plan for Recovery and Resilience. However, the target has not been achieved.

While the 30 MBPS coverage appears satisfactory, Italy is still behind in the Ultra-broadband (at least 100 MBPS), essential to fostering enterprises' digitalization. According to Istat, in 2019, 41% of firms with at least ten employed using a broadband connection (30 MBPS), and only 13.8% accessed 100 MBPS. The picture is made even more complicated by the geographical distribution of the Ultra-broadband, with "white area" (no coverage), "grey area" (only one provider), and "black area" (at least two providers); these areas are currently part of the Italian Strategy for Ultra-broadband that points to reducing the gap in the next three years. However, two out of three Italian SMEs are in the grey area where the ultra-broadband is practically missing. In 2019, Italy completed Phase I of the ultra-broadband Italian plan for white areas and awarded the last three tenders to the wholesale-only operator Open Fiber. Nevertheless, severe delays remain, mainly due to existing infrastructure and obtaining regional permits.

**Fig. 1:**
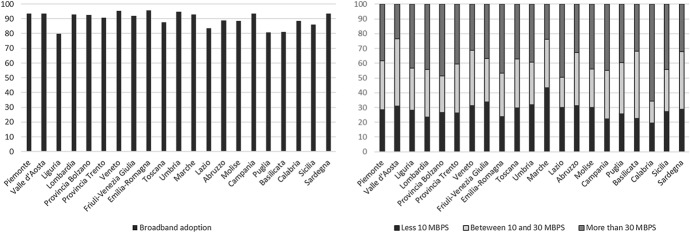
Firms' broadband adoption in 2019 (left). Type of connection in 2019 (right)

This paper focuses on net firms' broadband adoption (*Broadb*), rather than coverage. Istat provides regional data on the firms' network adoption since 2003, but only since 2019 by type of connection. Figure 1 shows the regional distribution of firms' broadband adoption (%) belonging to sectors from C to N, excluding K^2^ (on the left side). Given the regional-specific percentage of adoption, the multi-grey bars depict three types of connection: "less than 10 MBPS", "between 30 and 100 MBPS", and "more than 100 MBPS" (on the right side).

At first glance, the Southern regions show an adoption share not so distant from the North levels (except Puglia and Basilicata). In some cases, Southern areas show a high degree of ultra-broadband adoption (more than 100 MBPS)**.** However, this has not driven a consequent increase in productivity in Southern firms. As an example, let us focus on Calabria. This region is in the deep South of Italy, with a low average income, very high unemployment (particularly in the young), and coarse entrepreneurship. Despite this, it ranks at the top of the regions with firms connected in ultra-broadband (more than 100 MBPS) in 2019. So, for some regions, broadband adoption is not enough for boosting their economy. Is this a characteristic of Southern regions that cannot exploit this digital advantage for filling the productivity gap? Is this the lack of other dimensions, such as entrepreneurship, human capital, firms’ strategy? We try to answer by an empirical investigation. Unfortunately, before 2019, Istat did not provide detailed data by type of connection. The only regional data available concerns firms' share connected to a fixed network, from 2 MBPS, although the primary connection type is 30 MBPS.

## Estimating the TFP: some open questions

Our paper's first step involves estimating a traditional Cobb Douglas production function augmented by a Hicks neutral technical progress (TFP). The estimation of a production function is debated in the literature. Several approaches are used, from simple growth account decomposition of GDP to parametric estimates (since Solow, 1957 and its extension to cross-section and panel models), semi-parametric approach (Ackerberg et al., 2015; Levinsohn & Petrin, 2003; Olley & Pakes, 1996) until non-parametric methods such as the stochastic frontier approach and DEA, since the pioneering contribution of Aigner and Chu's (1968). However, the parametric approach is the most popular among researchers. Nonetheless, its reliability opens to significant debate, focused primarily on the endogeneity of inputs and productivity in the regression equation. In 1996, Olley and Pakes's contribution showed "the simultaneity bias" of the traditional regression model; they proposed a semi-parametric approach to overcome the question. A good survey of a parametric vs. semi-parametric approach is in Beveren (2012). Here we sketch the question.

Let us start by assuming that the market output of a representative firm *i* is given by a traditional production function, with Hicks-neutral technical change:

1 $${Y}_{i}(t)={A}_{i}(t)F({K}_{i}(t),{L}_{i}(t))$$

where *F*($.,.$) is a well-behaved production function whose inputs $K(t)$ and $L(t)$ are capital and labor; *A*(*t*) is the technical change (or TFP). Let us assume that $F(.,.)$ is a Cobb Douglas; in logs we have:

2 $${y}_{it}={\beta }_{0}+{\beta }_{l}{l}_{it}+{\beta }_{k}{k}_{it}+{\varepsilon }_{it}$$

where ${y}_{it}$ is the log of output, ${k}_{it}$ is the log of capital input, and ${l}_{it}$ is the log of labor input, all of which observable. $\mathit{ln}\;{A}_{it}={\beta }_{0}+ {\varepsilon }_{it}$ where ${\beta }_{0}$ measures the mean efficiency level across firms and over time; ${\varepsilon }_{it}$ is the time—and producer—specific deviation from that mean, which further decomposes into an observable ${v}_{it}$ (or at least predictable) and unobservable component ${u}_{it}$. We obtain the following equation:

3 $${y}_{it}={\beta }_{0}+{\beta }_{l}{l}_{it}+{\beta }_{k}{k}_{it}+{v}_{it}+{u}_{it}$$

where ${\omega }_{it}={\beta }_{0}+{v}_{it}$ represents firm-level productivity and ${u}_{it}$ is a i.i.d. component, measuring unexpected deviations from the mean due to measurement error, unexpected delays, or other external circumstances. Typically, empirical researchers estimate Eq. (3) and solve for ${\widehat{\omega }}_{it}={\widehat{\beta }}_{0}+{\widehat{v}}_{it}={y}_{it}-{\widehat{\beta }}_{l}{l}_{it}-{\widehat{\beta }}_{k}{k}_{it}$. Finally, the estimated productivity in level is obtained by ${\widehat{A}}_{it}=(\mathit{exp}({\omega }_{it})).$ This productivity measure is usually used to evaluate the influence and impact of various policy or covariates, as we will do in the next section.

The "endogeneity of input choice" or "simultaneity bias" arises because productivity shocks are potentially known to the firms when they make input decisions, such as the managerial ability or an expected shortage in intermediate inputs and so on. Hence, the inputs choice—${k}_{it}$and ${l}_{it}$—depend on ${v}_{it}$ and OLS estimates of ${\beta }_{k}$ and ${\beta }_{l}$ are inconsistent, because of the correlation between factor inputs and ${\omega }_{it}$.

Usually, researchers assume ${\omega }_{it}={\omega }_{i}$, and the firm observes ${\omega }_{i}$ before choosing inputs. In this way, standard fixed effect approaches produce consistent estimates for ${\beta }_{k}$ and ${\beta }_{l}$. Nevertheless, this implies that shocks are time-invariant, an extreme assumption. The question was firstly addressed by Olley and Pakes (1996—OP) and later by Levinshon and Petrin (2003—LP), by getting shocks to be time-variant and depending on capital input (OP) or in intermediate input (LP).

In OP ${v}_{it}={\Phi }_{t}({k}_{it},{i}_{it})$ where ${i}_{it}$ is the investment function so that $E({v}_{it}{k}_{it})\ne 0.$ LP use a different approach by starting from a production function augmented with the intermediate input ${m}_{it}$ and assuming that ${v}_{it}={\Phi }_{t}({k}_{it},{m}_{it})$ where ${m}_{it}$ are intermediate inputs. Both propose a two-stage approach to estimate parameters of Eq. (3). Nevertheless, both approaches treat the labour input choice as independent from the productivity shock, which seems a rather strong assumption. More recently, Ackerberg et al., (2015—ACF hereafter) show that such a two-stage approach implies what they call "functional dependence" in the first stage of OP and LP; being $l$ functionally related to $k$ via firms' first-order condition, this makes inconsistent the estimates of ${\beta }_{l}$ obtained in the first stage of these methodologies. Hence, they suggest an alternative method acting at estimating both ${\beta }_{l}$ and ${\beta }_{k}$ in the second stage (see Appendix A1).

As said, whenever we can assume ${\omega }_{it}={\omega }_{i}$ and the firm knows ${\omega }_{i}$ before choosing inputs, standard fixed-effect approaches produce consistent estimates for ${\beta }_{k}$ and ${\beta }_{l}$. Hence, the debate parametric vs. semi-parametric estimation lies in the assumptions on the shocks' nature. With regional, rather than firms, data, shocks are treated either as a random or fixed time-invariant component in a panel approach to capture regional heterogeneity (see, for example, Bronzini, & Piselli, 2009). Nevertheless, parametric estimators that do not control the spatial correlation produce biased results, and a spatial econometric approach is strongly advised.

Concluding, both approaches (semi-parametric vs. parametric) have lights and shadows for our aim. On one hand, the former is well-founded in the firms' behaviour while the latter is more suitable for regional data. For such a reason, in this paper, we present both estimation strategies. The semi-parametric approach uses the GMM approach by ACF, described briefly in Appendix A1, while the second exploits an ML estimator for panel data augmented by the spatial components. However, the results are pretty comparable.

## Filtering the TFP

In this section, we are going to estimate Eq. (3) both by the ACF approach and a spatial panel. As discussed in the previous section and Appendix A1, the ACF approach relies on a semi-parametric GMM on nominal GVA, using the GVA deflator and an intermediate input (firms’ electricity consumption) as instruments; the reason for using the GVA deflator lies in the “price-bias” question, as explained in Appendix A1. About the spatial error model (SEM), where the spatial autocorrelation is embodied in the error component, the choice is justified by its characteristics. The SEM model is advised "in the presence of a spatially dependent omitted variable that is correlated with the included explanatory variable" (LeSage & Pace, 2009). In our case, the SEM structure is a suitable choice, as the inputs (labour, and capital) could be related to other variables not specifically considered in the regression (such as human capital, intermediate inputs, and so forth). In our paper, ${y}_{i}$ is the (log)nominal gross value added of each Italian Region’s business sector (NUTS 2), where $i$ is the Region. For the reasons illustrated in Appendix A1, we use the nominal value as dependent and the deflator as an instrumental variable; deflator comes from the ratio of output in current and concatenated values.

Nevertheless, the Cobb Douglas estimate at the regional level involves the knowledge of labour and capital at NUTS2 level. Unfortunately, an official time series of the stock of capital at NUTS 2 does not exist, while the gross fixed investment measure at NACE level is available. So, following the Bank of Italy (Bronzini, & Piselli, 2009; Filippone, & Montanaro, 2014), we use a traditional Perpetual Inventory Method to estimate the private regional capital stock. Data comes from the Istat, over the period 2003–2018.

Once estimated the Cobb Douglas, the TFP is then calculated by the inverse logarithm of the residuals of the Eq. (3).

Tables 1 and 2 show the results for both estimation approaches of the Cobb Douglas.

**Table 1:** ACF GMM estimate

	Estimate	Std. Error	t value	Pr( >|t|)
logK	0.390	0.050	7.835	0.000
logL	0.669	0.089	7.555	0.000

	Statistics	df	p-value
J-Test E(g) = 0	0.344	1	0.558
W-Test	0.620	2	0.733

**Table 2:** ML SEM approach

	Estimate	Std. Error	t value	Pr( >|t|)
logK	0.522	0.031	16.832	0.000
logL	0.609	0.057	10.624	0.000
$\uplambda$	0.364	0.054	6.687	0.000
	Statistics	df	p-value	
W-Test	3.967	1	0.046	

	Statistics	df	p-value
W-Test	3.967	1	0.046

Both methodologies show significant and less than one coefficients of the production function (DRS on a single input). Moreover, the J test on the instruments’ validity of the GMM is successful. The sum of the estimated coefficients is slightly higher than one in both estimations (in particular for the ML estimation^3^). Nevertheless, the point estimates must not be considered a proof for the joint returns to scale. As known, to test the linear restriction H0: ${\beta }_{l}+{\beta }_{k}=1$ we must run a Wald test $.$ In the case of GMM approach, the test confirms that the assumption of constant return to scale is satisfied at least at 5% and that our theoretical model is coherent with the data. The ML model is slightly below the rejection threshold at 5%.

In general, the GMM model seems more reliable than the ML. This is because it allows controlling for the endogeneity effect, instruments are validated, and the Wald test is robust. For such a reason, we will use the TFP calculated by the GMM approach in the following analysis.

**Fig. 2:**
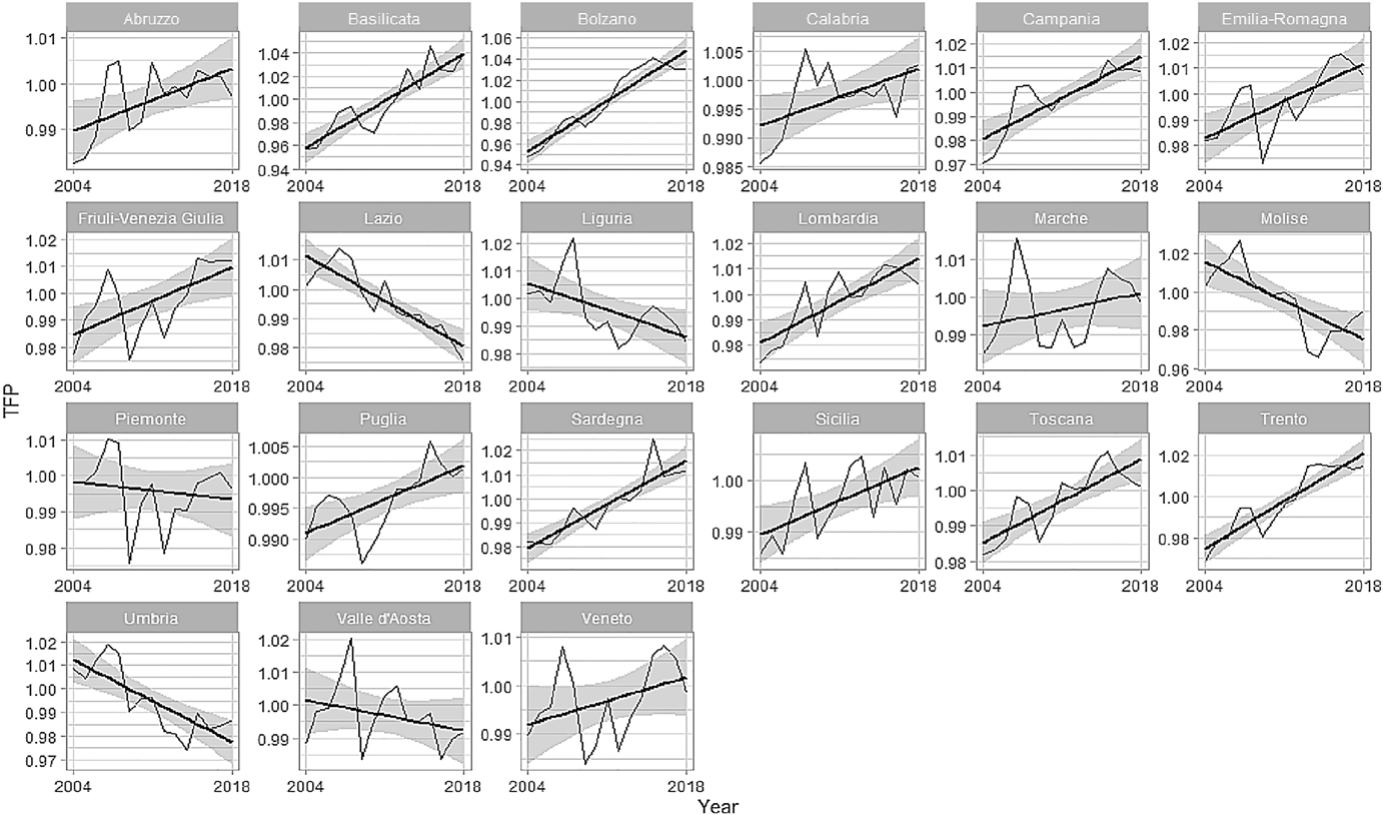
Estimated TFP

Figure 2 shows the estimated regional TFP; in some cases, it is upward sloping, but in some others, the path is reversed, confirming that Italian regions behave very differently from each other. There are specific regional effects that must be considered when using the TFP as an endogenous variable.

In Fig. 2, some results were largely expected. TFP is increasing in Lombardia, Emilia-Romagna, Trento, Bolzano, Friuli-Venezia-Giulia, and Toscana (all in the North). These regions are among the best performers in Italy in terms of private firms' productivity. More surprising is the good performance of some Southern regions, like Campania, Basilicata, and Sardegna. The remaining regions show either a severe loss in productivity or a stable value with fluctuations. Lazio is worth further analysis: its TFP is steadily decreasing, although it belongs to the high growth regions. However, Lazio's economy relies on the public rather than the private economy, and the latter suffered a slow decay over the last twenty years.

Regarding the statistical properties of the regional TFP, cross-dependency could be at work, as we expect spatial interdependence among Italian regions; for such a reason, we perform the Pesaran CD test^4^ (Pesaran, 2006). Moreover, unit-roots (non-stationarity) could exist as well; we use the Pesaran CIPS^5^ test (Pesaran, 2007) to investigate the question. Table 3 shows CIPS and CD tests on the TFP.

**Table 3:** CIPS and CD test

	CIPS	CD
Test	– 0.41	17.56
th	– 1.63	– 2.75

CIPS test is performed with maximum lag = 1. H_0_: homogeneous non-stationary. CD test’s H_0_: no cross-sectional dependence. Th. is the critical value at 5%

The CIPS test suggests the presence of unit root at 5 per cent, rejecting the stationarity assumption in TFP. Further, the CD test confirms a high degree of cross-sectional dependence and confirms regional spillovers exist.

## ICT and TFP

Once having filtered the TFP, we can empirically assess how firm investments in the digital economy impact the regional technical change.

The presence of a unit root and stochastic trend in the TFP may suggest a possible long-run relationship with our main variable, the broadband adoption, in case the latter owns a unit root as well. Following the Engle and Granger (1987) approach, we will assess whether a cointegration equation exists among these two variables. If so, the Engle and Granger theorem points out an Error Correction Model (ECM) estimation strategy.

Some covariates may augment the ECM. The literature identifies different elements which might affect the TFP at the local level. As underlined in Bugamelli et al. (2018), the productivity growth in Italy can be driven by innovation and technology, human capital, competition, regulation, and so on.

In this context, we select the following variables as further drivers of the relationship between TFP and broadband adoption:

1. *Pc* – Percentage of firms using a PC—Business Sector
2. *Int* – Percentage of employees using Internet facilities—Business Sector
3. *Stem*—Share of graduates in Stem disciplines
4. *FI*—log of Private Investment—share on GDP
5. *Human Capital* – Population with tertiary education
6. *Internationalization*—Degree of internationalization
7. *High-tech enterprises*—Birth rate of knowledge-intensive enterprises

The first variables (*Pc* and *Int*) and broadband adoption cover the connection and the adoption of digital solutions. As previously discussed, we are interested in the first two DESI components and, partly, in point 4—connection, human capital, and digital economy integration in the Italian firms. *Stem* and the population with tertiary education account for the human capital. Moreover, productivity is also affected by physical investment (*FI*), which can be considered a proxy for firms' competitiveness. Finally, the degree of internationalization and number of newborn firms in knowledge-intensive sectors are further controls that could impact the TFP of private firms. Data are provided by Istat that collects, since 2003, information at NUTS2 level on a series of variables related to statistics for the local development and social cohesion. In particular, the section Information Society records data on the digital economy for firms with more than ten employed.

Besides these, several forces affect local productivity, related to the economic tissue and the social, demographic, legal, and environmental context. Each region has its peculiarities regarding goods specialization, firms' characteristics, average standard of living, social and cultural capital, etc. These factors are related to regional-specific components and their heterogeneity. The fixed effects estimator can capture the latter in the empirical model, which accounts for specific regional components not taken into specific view by exogenous variables.

Moreover, as previously stressed, technology spreads over time and space. Regional spillovers could be at work, which must be considered in the empirical analysis. The spatial dimension allows us to suggest local development policies addressed to narrow local gaps.

However, the direct use of all the covariates in a regression equation could bring to a collinearity problem (endogeneity), as they are complementary and related to each other, especially Broadb, Pc and Int.

To clarify the question, let us start from a general equation for the TFP model (later, we will see the diff version in Eq. 4):

$${TFP}_{it}={\alpha }_{0}+{\alpha }_{1}{Broadb}_{it}+{\alpha }_{2}{X}_{it}+{\mu }_{i}+{\varepsilon }_{it}$$

where *i* is the i-th region, *t* the time, *X* a matrix of exogenous variables and ${\mu }_{i}$ the individual effect. To consistently estimate the equation by OLS or LM, the Gauss Markov theorem requires the orthogonality condition $E\left(Broad{b}_{it}\cdot {\varepsilon }_{it}\right)=0$. The innovation component ${\varepsilon }_{it}$ must strike only the left-hand side of the equation. Nevertheless, if ${\varepsilon }_{it}$ were due to a technological shock, likely it would induce firms to reconsider their ICT investment, including the adoption of a broadband infrastructure, and the orthogonality condition would be violated. In this situation, the instrumental variable (IV) technique must be applied to recover estimates consistency. As known, this approach manages the endogeneity question by introducing additional variables (instruments) which are correlated with the suspect endogenous variable $Broad{b}_{it}$ but not with ${\varepsilon }_{it}.$ The IV estimator is analogous to a two-stage approach: in the first one $Broad{b}_{it}$ is regressed on the instruments; then, in the second stage, the fitted values $\widehat{B}road{b}_{it}$ are used in the TFP equation, as $E\left(\widehat{B}road{b}_{it}\cdot {\varepsilon }_{it}\right)=0$. This opens to the difficult task to select the instruments for Broadb. There is not a “golden rule”, but some “rules of thumb” based on the vast empirical literature. One of these, is to look at the correlation matrix of the variables.

We wonder whether Pc and Int could be used for explaining Broadb. Nonetheless, the shock striking the latter could hit also the former, arising the endogeneity question once again. To have a first clue at the joint relationship among the variables, Table 4 shows the correlation matrix:

**Table 4:** Correlation matrix

	Broadb	Pc	Int
Broadb	1		
Pc	0.73	1	
Int	0.35	0.65	1

The highest correlations are between Pc and Broadb, and between Pc and Int. The correlation between Broadb and Int is definitely lower. Hence, Table 4 suggests trying Pc as an endogenous variable for Broadb (as they are correlated) and Int as instrument for Pc, as the latter is weakly correlated to Broadb but highly correlated to Pc. The instruments validity will be then checked by the F-test in Table 5.

**Table 5:** IV estimates

Dependent variable		
	${\mathrm{Pc}}_{\mathrm{it}}$	${\mathrm{Stem}}_{\mathrm{it}}$
${\mathrm{Broadb}}_{\mathrm{it}}$	1.291***	8.767**
	(0.308)	(0.518)
Observations	315 Yes 35	
FE F-test		

S.E. in parentheses; *p < 0.10; **p < 0.05; *** p< 0.01

Summing up, we have a recursive endogeneity problem. Broadb is an endogenous variable for the TFP equation, but Pc is endogenous for Broadb. To solve the problem, we start from the “bottom” by using the IV estimator to estimate consistently the Broadb equation, using Int as an instrument for Pc. Once estimated the Broadb, the fitted values can be used as regressor in the TFP equation, as $E\left(\widehat{B}road{b}_{it}\cdot {\varepsilon }_{it}\right)=0$, solving the endogeneity question.

In the following section, we start from applying the IV approach to the variables Broadb, Pc and Int.

### The IV estimation for broadband adoption

As said, before estimating our ECM, we perform a linear model using an instrumental variables (IV) approach, with fixed effect. We regress Broadb on Pc and Stem, using Int as an instrumental variable for Pc. Stem is treated as exogenous variable, but it could be helpful in gaining efficiency of the Broadb estimate. Results are in Table 5.

 PC and Stem positively impact the firms' broadband adoption through the Internet facilities, as expected. In addition, the growing share of graduates in Stem disciplines in most regions has effectively encouraged companies to use ICT instruments and adopt broadband. The F-test on the instrument validity is higher than 10, confirming that Int can be treated as an instrumental variable.

Once having estimated Broadb in a consistent way, the fitted values will be used as a covariate in the TFP equation for the reasons previously stressed. This is performed in the following section.

### TFP and broadband: cointegration analysis

According to Engle and Granger (1987), if two series are non-stationary (i.e. I(1)), but a linear combination of them is stationary, the two variables are cointegrated, following a common long run relationship.

To investigate the question, we must firstly check the unit root both in TFP and in the fitted value of the IV estimation, $\widehat{B}roadb$. The CIPS test in Table 6 confirms that TFP and broadband are non-stationary. Then, we must verify that both equations are cointegrated, which means that a linear combination of the two variables produces a stationary residual. Following Engle and Granger (1987), we estimate the following linear combination:

**Table 6:** CIPS test

	${\mathrm{TFP}}_{\mathrm{it}}$	$\widehat{\mathrm{B}}{\mathrm{roadb}}_{\mathrm{it}}$	${\widehat{\mathrm{u}}}_{\mathrm{it}}$
Test	0.41	1.20	– 1.97
th	– 1.63	– 1.63	– 1.63

CIPS test H_0_: homogeneous non-stationary. Th. is the critical value at 5%

$${TFP}_{it}-\gamma \widehat{B}road{b}_{it}+{\mu }_{i}={u}_{it}$$

where $i$ is the region, $t$ the time, ${\mu }_{i}$ is the regional specific effect (fixed effect), and ${u}_{it}$ is the error term of the cointegration’s relationship. The ML estimator with fixed effect produces $\widehat{\gamma }=3.17 {10}^{-4}$ with a p-value of 7.22 ${10}^{-10}$ hence statistically significant. To check the cointegration relationship, the estimated residual component $\widehat{u}$ must be a stationary random process; the right column of Table 6 shows that the CIPS test reject the null of unit root.

The cointegration equation acts as a long run constraint between the TFP and the broadband adoption in an empirical model. Engle and Granger (1987) show that the two variables adjust over time to the long run constraint $\widehat{u}$, following an error correction model (ECM). The latter is composed of a short-run dynamics (the variables in first difference, hence stationary) which adjusts to the long run constraint ${\widehat{u}}_{it-1}$ with a temporal lag. According, we estimate the ECM as below:

4 $$\Delta {TFP}_{it}=\pi {\widehat{u}}_{it-1}+{\beta }_{1}\Delta \widehat{B}road{b}_{it}+{\beta }_{k}\Delta {X}_{it}+{\mu }_{i}+{\varepsilon }_{it}$$

where $\pi$ is the adjustment coefficient to the long run constraint. It measures the speed of adjustment to the long-run equilibrium. The adjustment coefficient must be smaller than one and negative to converge to the steady-state path. ${X}_{it}$ is the vector of covariates that control for competitiveness, human capital, and internationalization. ${\varepsilon }_{it}$ is i.i.d disturbance with mean 0 and homoscedastic variance ${\sigma }^{2}$. $N=21$ is the number of Italian regions^6^ an $T=16$ years (2003–2018); consequently, the panel in first differences consists of 294 observations.

We estimate the ECM by a ML panel with fixed effect (results are in Table 7 left column), but the residual component is affected by cross-sectional dependence, as the CD test shows. We augment the baseline ECM with a spatial lag to account for the cross-sectional dependence. A spatial autoregressive model (SAR) is performed as follow:

**Table 7:** ECM estimates

	Dependent variable	
	(1)	(2)
	$\Delta$ TFP	$\Delta$ TFP
${\uppi \widehat{\mathrm{u}}}_{\mathrm{t}-1}$	– 0.227***	– 0.178***
	(0.037)	(0.031)
$\Delta \widehat{\mathrm{B}}\mathrm{roadb}$	0.0002*	0.0001
	(0.0001)	(0.0001)
$\Delta$ FI	0.059***	0.031***
	(0.014)	(0.012)
$\Delta \mathrm{H}$ uman capital	0.016*	0.015**
	(0.009)	(0.007)
$\Delta$ Internationalization	0.001***	0.001***
	(0.0002)	(0.0002)
$\Delta \mathrm{K}$ nowledge-intensive enterprises	0.001***	0.0005*
	(0.0003)	(0.0003)
$\uprho$	–	0.430***
		(0.049)
Observations	294	294
FE	Yes	Yes
CD test	14.70	– 1.41
th	– 2.75	– 2.75

S.E. in parentheses; *p < 0.10; **p < 0.05; ***p < 0.01. CD test’s H_0_: no cross-sectional dependence. Th. is the critical value at 5%

4b $$\Delta {TFP}_{it}=\rho W\Delta {TFP}_{it}+\pi {\widehat{u}}_{it-1}+{\beta }_{1}\Delta \widehat{B}road{b}_{it}+{\beta }_{k}{X}_{it}+{\mu }_{i}+{\varepsilon }_{it}$$

where $\rho$ the spatial autoregressive coefficient, $W$ the row standardized spatial matrix with one if the regions are neighbours and zero otherwise.

The choice of the SAR model, instead of a Spatial Error Model (SEM), is justified by the different hypothesis about spatial externalities. In the SEM case, spatiality in the error component captures possible common factors and possible cross-dependence in omitted variables. In a SAR model, spatial correlation arises through externalities related to the endogenous variable. Such an assumption fits our basic assumption: the TFP embodies ideas and knowledge that are spread in the economy; it generates positive spillovers that are beneficial also for others.

The results are in Table 7, where columns (1) and (2) show the panel linear and SAR model, respectively. As we use a variable coming from the IV stage (Broadb), the standard errors in the second stage are obtained by bootstrapping over 400 repetitions. According to the Pesaran’s CD test (in Table 7), we cannot reject the assumption of cross correlation in non-spatial ML residuals. Differently, the ML spatial estimation coefficients are unbiased because the test suggests no cross-sectional dependence.

The spatial autoregressive coefficient is relatively high ($\rho =0.43$) and confirms the role of spatial spillovers in the estimation.

It is important to note that, as shown in LeSage and Pace (2009), spatial models require a different interpretation of estimated coefficients than the non-spatial model. The spatial spillovers embodied in the spatial model requires a different way at assessing how changes in the explanatory variables affect the endogenous variable; for such a reason, the estimated coefficients of the right column of Table 7 are not interpretable. Broadly speaking, a change in the explanatory variables, in region *i,* impacts not only on the endogenous variables in the *i* region itself but on neighbours as well, and, for a feedback effect, from the neighbours to the region *i* (the so-called “global spatial spillovers”). The impact of the covariate is hence divided in indirect and direct impacts; the first one accounts for the effect on region *j* of a change in region *i*, while the second one of the region *i* on the region itself. The sum of direct and indirect impacts produces the total impact, providing the right estimate coefficient measure. Each impact’s statistical significance must be calculated using a bootstrap algorithm, as discussed in LeSage and Pace (2009).

Table 8 shows direct, indirect, and total impacts. The error correction term is negative and significant. So, the ECM converges to the long-run equilibrium. For what concerns covariates, direct and total impacts show a positive sign, as expected. In magnitude, the direct impacts are similar to the estimated coefficients of the spatial model. Moreover, the results confirm our hypothesis of a positive relationship between broadband and TFP. In fact, the adoption of digital technologies affects directly and positively the productivity in Italian regions. Fixed investments, human capital, internationalization, and new enterprises, boost regional productivity growth as well.

**Table 8:** Spatial impacts

	Direct	Indirect	Total
${\uppi \widehat{\mathrm{u}}}_{\mathrm{t}-1}$	– 0.189***	– 0.122***	– 0.312***
$\Delta \widehat{\mathrm{B}}\mathrm{roadb}$	0.0001*	0.0001	0.0002*
$\Delta$ IF	0.034***	0.022**	0.055***
$\Delta \mathrm{H}$ uman capital	0.017**	0.011*	0.028**
$\Delta$ Internationalization	0.001***	0.0005***	0.001***
$\Delta \mathrm{K}$ nowledge-intensive enterprises	0.001*	0.0003*	0.001*

S.E. in parentheses; * p < 0.10; ** p < 0.05; *** p < 0.01

Additionally, as underlined by indirect effects, the model captures the presence of spatial spillovers.

## Discussion and policy implications

**Fig. 3:**
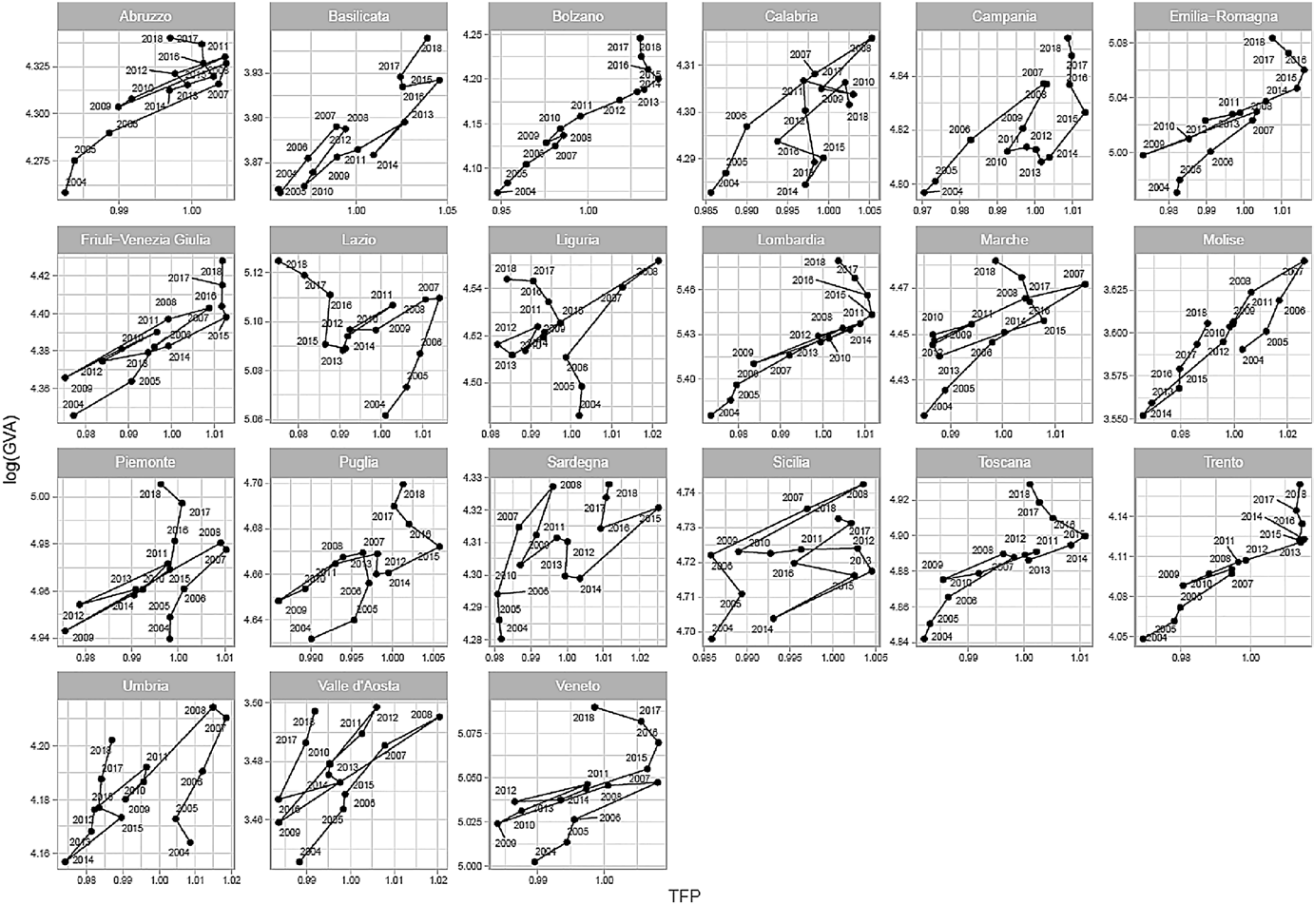
log GVA vs. TFP

We started our contribution by estimating the TFP of the Italian regional business sector. Figure 2 shows the remarkable differences among the regions. The TFP is, in some sense, an "obscure" object, as it comes from the residuals of a regression. For its nature, it embodies all that we do not know nor control. Moreover, from this point of view, it is a powerful source of information. In our results, according to the theory of economic growth, the TFP drives the firms' value-added; Fig. 3 shows the scatter plot between the TFP, on the horizontal axis and the log of GVA on the vertical one.

**Fig. 4:**
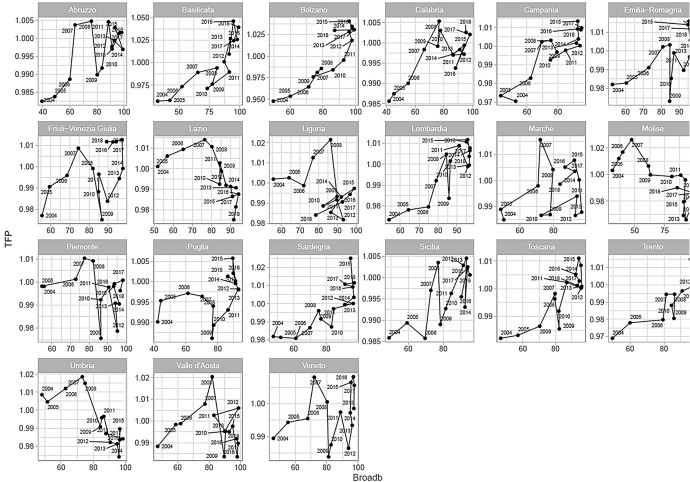
TFP vs. Broadband

**Fig. 5:**
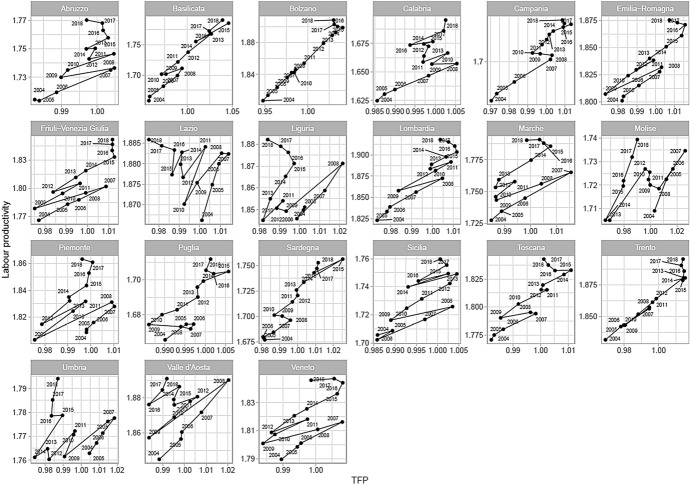
Labour productivity vs. TFP

From Fig. 3, until 2008, the GVA follows the behaviour of the TFP. Any time the TFP grows so does the GVA and vice versa. Nevertheless, the 2007/2008 crisis breaks such a positive relationship, as it works as a turning point. After 2008, the positive relationship between TFP and GVA gets less readable in almost all Italian regions. From 2008 and on, the relationship shows slumps and upswings with remarkable differences among the regions. Nevertheless, these alternate phases still show a positive relationship; GVA and TFP grow or reduce following each other. The 2008 structural change is well evident in Fig. 4 (in Appendix) also, where we report the dynamics of firms' broadband diffusion and TFP, and in Fig. 5 (in Appendix), which shows the relationship between TFP and labour productivity. Some regions were able to recover after 2014, with higher TFP and GVA than in 2004. Some others were not, ending in 2018 with a lower, or equal, value of the TFP but in general with a higher GDP. In the last three years, the relationship between GVA and TFP has been zero or has become negative due to a slowdown in TFP.

The recovery after 2014 differs from region to region. Table 9 shows some descriptive statistics about the growth rates of relevant variables, providing clues for interpreting our results. According to Table 9, GVA grew in all regions after 2014. In general, the Centre and Northern regions (Liguria excepted) show a higher GVA growth rate than the Southern regions, which does not translate into an increase in TFP. The GVA growth in the Centre and Northern regions (2.53% in average) has been associated with an increase in employment level (1.53% in average) and, in some cases, labour productivity. On the other hand, the Southern regions experienced, with some exceptions, a lower growth with increasing broadband adoption and a less evident drop in TFP (−0.09% on average). For instance, in Lazio, rising employment generates high levels of GVA, against a clear fall in TFP. On the contrary, Calabria shows a positive variation of GVA, TFP, and broadband adoption, and a fall in employment level. Moreover, the increase in broadband adoption has contrasted the fall in the TFP of Southern regions, especially between 2015 and 2018. In fact, Southern regions are more reactive in broadband adoption than Northern regions. As we underlined in our estimation, leaving aside the fixed effect $\mu$, a 1 point of increase in broadband usage increases firms' TFP by $2\cdot 1{0}^{-4}$. After all, it could seem a marginal effect but it is not, as variables are in logs and diffs. The same picture is in Fig. 4, where the increase in broadband usage has tempered the fall of the TFP after 2008.^7^

**Table 9:** Annual growth rate by period (percentage)

Region	TFP			GVA			Broadb			Employment			Labour productivity		
	04–08	09–14	15–18	04–08	09–14	15–18	04–08	09–14	15–18	04–08	09–14	15–18	04–08	09–14	15–18
Piemonte	0.28	0.31	– 0.06	2.39	0.74	2.82	10.99	2.22	1.28	1.06	– 0.76	1.27	1.31	1.53	1.53
Valle d'Aosta	0.80	0.23	– 0.19	2.90	0.81	1.92	15.23	1.18	0.90	0.26	– 0.23	1.53	2.64	1.02	0.37
Liguria	0.49	0.00	– 0.44	3.86	– 0.09	1.44	11.63	0.75	– 7.31	2.59	– 0.82	0.59	1.26	0.75	0.85
Lombardia	0.80	0.46	– 0.26	3.44	1.08	2.81	11.94	2.48	0.29	0.56	– 0.77	1.67	2.87	1.87	1.12
Bolzano	0.99	1.13	– 0.34	3.75	2.79	3.57	12.57	5.91	– 2.96	1.45	0.14	2.82	2.28	2.66	0.73
Trento	0.65	0.70	0.01	3.06	1.61	3.27	15.39	2.43	0.23	1.53	– 0.12	1.95	1.50	1.74	1.30
Veneto	0.28	0.27	– 0.26	2.53	0.92	2.72	15.10	3.24	0.64	1.27	– 0.23	1.96	1.24	1.15	0.75
Friuli-Venezia Giulia	0.56	0.49	– 0.03	3.05	0.81	2.34	10.33	2.45	– 2.78	1.30	– 1.07	0.89	1.72	1.90	1.44
Emilia– Romagna	0.53	0.66	– 0.21	3.48	1.85	2.86	12.80	2.63	1.02	1.64	– 0.20	1.73	1.80	2.06	1.11
Toscana	0.36	0.46	– 0.33	2.81	0.91	2.25	14.82	3.13	– 0.30	1.35	– 0.88	1.48	1.43	1.81	0.76
Umbria	0.16	– 0.34	– 0.09	2.95	– 1.05	2.24	11.98	2.63	– 2.91	2.41	– 1.04	1.04	0.53	0.00	1.19
Marche	0.49	0.27	– 0.30	3.01	0.18	2.01	13.59	4.78	– 2.91	1.28	– 1.27	1.64	1.70	1.48	0.36
Lazio	0.24	– 0.15	– 0.38	2.79	– 0.34	2.66	11.66	1.15	0.27	1.82	– 0.69	1.99	0.95	0.36	0.66
Abruzzo	0.56	0.14	– 0.20	4.03	0.44	1.58	18.08	4.36	– 0.66	2.71	– 0.50	0.61	1.30	0.93	0.96
Molise	0.09	– 0.68	0.36	2.00	– 2.44	2.95	18.58	8.55	– 1.54	1.40	– 1.80	1.40	0.58	– 0.67	1.53
Campania	0.83	0.14	– 0.15	2.36	– 0.49	2.14	11.67	3.54	0.75	– 0.52	– 1.61	1.79	2.89	1.14	0.35
Puglia	0.10	0.27	– 0.15	2.10	0.59	2.13	16.83	3.72	– 1.13	1.67	– 0.57	1.57	0.44	1.16	0.55
Basilicata	0.95	0.68	– 0.20	2.39	0.61	2.25	25.68	3.89	1.52	– 0.73	– 1.92	1.81	3.13	2.57	0.45
Calabria	0.50	– 0.04	0.11	1.91	– 0.93	0.89	16.56	2.86	0.72	0.03	– 1.71	– 0.55	1.89	0.82	1.47
Sicilia	0.45	0.08	– 0.06	2.60	– 0.88	1.27	12.21	3.40	0.52	1.43	– 1.96	0.47	1.19	1.11	0.81
Sardegna	0.36	0.24	– 0.44	2.74	– 0.60	0.57	12.34	5.01	0.89	1.62	– 2.12	0.83	1.11	1.54	– 0.25
**North-Centre**	**0.51**	**0.35**	**– 0.22**	**3.08**	**0.79**	**2.53**	**12.92**	**2.69**	**– 1.12**	**1.43**	**– 0.61**	**1.58**	**1.63**	**1.41**	**0.94**
**South**	**0.48**	**0.11**	**– 0.09**	**2.52**	**– 0.46**	**1.72**	**16.49**	**4.42**	**0.14**	**0.95**	**– 1.52**	**0.99**	**1.57**	**1.08**	**0.73**

Regarding the GVA growth, the crisis impacts more in the South, but the recovery of these regions has been greater than in the North due to a lower drop in TFP supported by broadband adoption (see Table 9 again). Although the GVA regional divide has not been closed at the end of 2018, if Southern companies, which could cable more, had not gained from digitalization, the divide would have been more severe. In this sense, broadband has played an important role as a shock absorber during the recession and tempered, in part, the North–South divide.

In conclusion, our results emphasize the digital economy's decisive role for Italian firms; moreover, this is true for Northern but more for Southern firms. The digitalization, through the TFP, positively impact on GVA recovery of Southern regions in recession periods. For this reason, going on with the digitalization of southern firms is a fundamental long-term policy action for filling the gap with the North. However, policymakers should also promote local development through employment and entrepreneurship policies to fill this historical gap.

## Conclusions and further research

This paper investigates the effect of the digital economy on TFP, notably related to broadband diffusion among private firms. The spatial econometric analysis confirms a positive relationship, and, under this point of view, it seems evident that policies fostering broadband, pc, and internet adoption in the Italian firms favour the local development.

The financial crisis broke a well-established pattern, as Fig. 4 in Appendix A1 shows. Before 2008, TFP and broadband followed an evident positive trend in all the regions, but data were more volatile after the crisis. Hence, the crisis was a severe structural change, and regional responses were somewhat scattered; some regions showed partial recovery, while others did not. For most regions, the increase in broadband usage has tempered the fall of the TFP. Especially in Southern regions, the increase in broadband usage has contrasted with the fall in the TFP and eventually triggered a positive trend. This is a hard lesson even for the current Covid-19 crisis that, unfortunately, will be much more dramatic for the Italian economy, and not only, than the 2008 one. Since 2014, regions have been showing a recovery trend. This recovery for the Northern regions is mainly linked to the improvement in employment and labour productivity levels. By contrast, in the Southern regions, the expansion of TFP, also driven by a high level of firms’ broadband adoption, has supported the growth of GVA. In this regard, the broadband adoption has played an important role as a shock absorber during the recession. Nevertheless, our results show that Italian regions are still characterized by heterogeneity. Although it was accounted for in the empirical model, the question of local disparities remains on the policymakers' agenda. Not only a North–South divide but a more complex picture of local interrelationships among clusters. The Recovery Plan in part fill the gap, as actions are diversified between North and South, but much of the story will be written in the territories and by local policymakers.

The digital economy has a decisive role for Italian firms. As our findings emphasize, the North–South divide seems to have been tempered by the digital economy and its impact on TFP. For this reason, going on with the digitalization of southern firms is a fundamental long-term policy action for filling the gap with the North. However, as pointed out in the introduction, the digital economy is only the sharp of an iceberg. It involves government actions (in terms of strategies for digitalization), firms' entrepreneurship (in terms of R&D and ICT investment, managerial organization, and business model), and human capital (in terms of education and training both in the youngest and the workers). The policymakers should carry out employment and entrepreneurship policies to make regions more resilient to external shocks and reduce the North–South divide. The ICT facilities are not enough for the Southern economies that need a boost in employment by creating new businesses. Therefore, within the Recovery Plan framework, the pillar of Social Inclusion is also crucial for sustainable development, especially in Southern regions.

## Figures and Tables

**Fig. 6:**
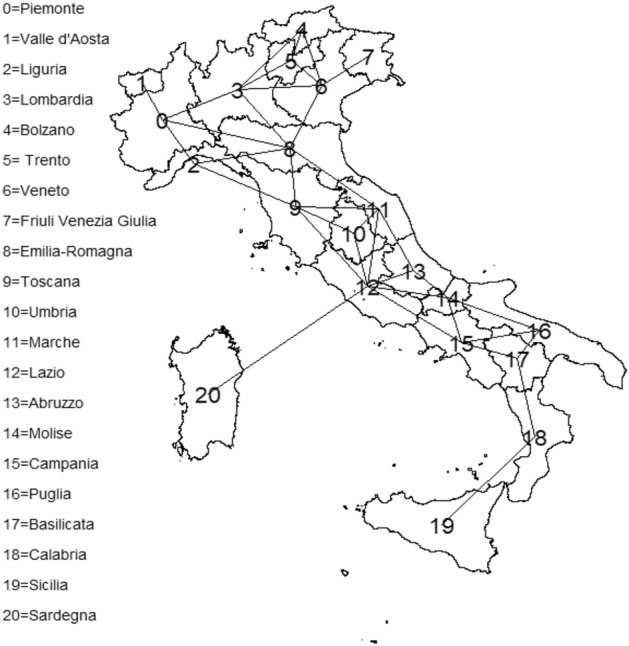
Italian regions and connectivity map

## Appendix

See Figs. 4, 5 and 6.

### A1. The GMM approach to TFP

We sketch the ACF approach here. Let us start from Eq. (3) in gross value units. The capital input evolves according to the traditional investment rule:

$${k}_{it}=f({k}_{it-1},{i}_{it-1})$$

where the investment ${i}_{it-1}$ is known at time $t-1$, consequently ${k}_{it}$ is known as well at time $t-1.$ The demand for intermediate inputs ${m}_{it}$ depends on the choice of the inputs and productivity shocks:

$${m}_{it}=g({k}_{it},{l}_{it},{v}_{it})$$

in LP the intermediate demand is not conditional on labour input; this is the main difference. By inverting the intermediate inputs demand, we obtain the productivity shock ${v}_{it}={g}_{t}^{-1}({k}_{it},{l}_{it},{m}_{it})$ that can be plugged into Eq. (3):

5 $${y}_{it}={\beta }_{0}+{\beta }_{l}{l}_{it}+{\beta }_{k}{k}_{it}+{g}_{t}^{-1}({k}_{it},{l}_{it},{m}_{it})+{\varepsilon }_{it}$$

this makes the first three terms on the rhs not identified.

By letting

6 $${\varphi }_{t}({k}_{it},{l}_{it},{m}_{it})={\beta }_{0}+{\beta }_{l}{l}_{it}+{\beta }_{k}{k}_{it}+{v}_{it}$$

we can rewrite Eq. (5) as:

7 $${y}_{it}={\varphi }_{t}({k}_{it},{l}_{it},{m}_{it})+{\varepsilon }_{it}$$

so, as the usual condition of orthogonality $E({\varepsilon }_{it}|{I}_{t})=E({y}_{it}-{\varphi }_{t}({k}_{it},{l}_{it},{m}_{it})|{I}_{t})=0$ must hold. As in OP and LP, the function ${\varphi }_{t}({k}_{it},{l}_{it},{m}_{it})$ can be approximated by a third order polynomial, so as Eq. (7) can be easily estimated by OLS to obtain the estimate ${\widehat{\varphi }}_{t}({k}_{it},{l}_{it},{m}_{it});$ this is the first stage of ACF.

Being ${v}_{it}$ a first-order Markov process, by assumption, in OP, LP and ACF, this can be modelled by an AR(1) ${v}_{it}=\rho {v}_{it-1}+{\xi }_{t},$ with $\rho <1$ and ${\xi }_{it}\sim n.i.i.d.(0,{\sigma }^{2})$ white-noise process. Equation (3) can be hence rewritten as:

8 $${y}_{it}={\beta }_{0}+{\beta }_{l}{l}_{it}+{\beta }_{k}{k}_{it}+\rho {v}_{it-1}+{\xi }_{t}+{\varepsilon }_{it}$$

By Eq. (6) ${v}_{it-1}={\varphi }_{t-1}({k}_{it-1},{l}_{it-1},{m}_{it-1})-{\beta }_{l}{l}_{it-1}-{\beta }_{k}{k}_{it-1}.$ By plugging the latter in Eq. (8), leads to:

9 $${\xi }_{t}+{\varepsilon }_{it}={y}_{it}-{\beta }_{l}{l}_{it}-{\beta }_{k}{k}_{it}-\rho \left({\varphi }_{t-1}({k}_{it-1},{l}_{it-1},{m}_{it-1})-{\beta }_{l}{l}_{it-1}-{\beta }_{k}{k}_{it-1}\right)$$

where ${\varphi }_{t-1}$ is replaced by the first-stage estimate ${\widehat{\varphi }}_{t-1}$. In order to estimate the model parameters, we have to impose $E({\xi }_{t}+{\varepsilon }_{it}|{I}_{t-1})=0.$ However the model has four parameters, ${\beta }_{0},{\beta }_{l},{\beta }_{k}$ and $\rho ,$ so we need at least other four moments condition. ACF, in the second stage, propose the following conditions to identify exactly the model:

$$E\left[\left({\xi }_{it}+{\varepsilon }_{it}\right)\otimes \left(\begin{array}{c}1\\ {k}_{it}\\ {l}_{it-1}\\ {\widehat{\varphi }}_{t-1}({k}_{it-1},{l}_{it-1},{m}_{it-1})\end{array}\right)\right]=0$$

where $\left({\xi }_{it}+{\varepsilon }_{it}\right)$ is given by Eq. (9).

We have four conditions in the moments that correctly identify the four parameters. However, as stressed by ACF, other conditions can be added depending on the input choice assumed timing. Investment at time $t$ could be a further instrument if ${i}_{it}\in {I}_{t-1},$ or the labour input ${l}_{it}:$ "In some industries, one might be willing to assume that labor is chosen by the firm at $t-1$, that is, ${l}_{it}\in {I}_{t-1}$ (or alternatively make the assumption that ${\omega }_{it}$ is not observed by the firm until period t + 1)". The latter is a potentially strong assumption, but it might be plausible when there are significant hiring or firing costs, or labour market rigidities, possibly due to government regulation. This stronger assumption will generally lead to more precise estimates" (ACF, page 2430). By adding other instruments, the model is over-estimated and the GMM can be implemented, testing for restrictions.

The estimated $log(Tfp)$ is finally obtained by

$${\widehat{A}}_{it}={\widehat{\beta }}_{0}+{v}_{it}={y}_{it}-{\widehat{\beta }}_{l}{l}_{it}-{\widehat{\beta }}_{k}{k}_{it}$$

and in levels by $TF{P}_{it}=\mathit{exp}({\widehat{A}}_{it}).$

However, the simultaneity bias is not the only open question. The multi-product firm, firm entry and exit choice, and perfect vs. imperfect markets represent other biased channels. In particular, the "price-bias" is relevant for our analysis. The theoretical model assumes that variables are in physical units, which makes impossible an empirical analysis. Had the output and input prices known for each firm, one could deflate nominal values, but these data are rarely available. Very often, researchers use average deflators as the industry one instead of single firm prices. However, firms' prices are correlated with input choices, causing once again a simultaneity bias. We can easily show this. Now, let us assume that inputs are in real terms, so the problem concerns only the output variable. If output ${z}_{i}$ is in value $({z}_{i}={p}_{i}{y}_{i}),$ and we use the average industry deflator ${{p}}_{i}$ instead of firm price ${p}_{i},$ the deflated output value is ${z}_{i}/{{p}}_{i}=({p}_{i}{y}_{i})/{p}.$ In log we get ${r}_{i}=\mathit{log}({z}_{i}/{{p}}_{i})=\mathit{log}{y}_{i}+\mathit{log}{p}_{i}-{{\mathit{log}p}}_{i},$ where $\mathit{log}{y}_{i}$ is given in Eq. (3). Hence, by deflating by industry deflator, brings to estimate the following equation:

$${r}_{it}={\beta }_{0}+{\beta }_{l}{l}_{it}+{\beta }_{k}{k}_{it}+{v}_{it}+{u}_{it}+\mathit{log}{p}_{i}-log\overline{{p }_{i}}$$

Now, being firm price unobservable (hence embodied in the error term), and the input choice affected by these prices, the orthogonality condition between covariates and error term is no longer satisfied, leading to estimates bias.

Even worse, when the capital stock is also in nominal value (as usual), using some deflated value brings further bias in the estimate.

The price bias in an unescapable question unless firm prices are available, but this is very rare. A possible way out is to consider the deflator, or generally known prices, as an instrumental variable in IV or GMM estimators in the estimated equation and testing for instruments validity (J-test) (see Ackerberg et al., 2007). We will follow this approach.

### A2. Pesaran CD and CIPS test

In this appendix, we review some "tools" of spatial econometrics. Let us start from the cross-dependency test by Pesaran (2006).

To test for cross-dependency, we use a CD test. The test is "is applicable to a variety of panel data models including a stationary and unit-roots dynamic heterogeneous panel with structural breaks with short T and large N. The CD test is based on the pair-wise correlations of the OLS residuals from the individual regressions in the panel and tends toward a standard normal distribution under the null hypothesis of no error cross-sectional dependence". (Holly et al., 2010, p. 164. HPY hereafter).

Generally, the Pesaran CD test is the following:

$$CD=\sqrt{\frac{2T}{\left(N-1\right)}}\left(\sum_{i=1}^{N-1}\sum_{j=i+1}^{N}{\widehat{\rho }}_{ij}\right)$$

where ${\widehat{\rho }}_{ij}$ is the sample estimate of the pair-wise correlation of the residuals coming from the estimates under hypothesis:

$${\widehat{\rho }}_{ij}={\widehat{\rho }}_{ji}=\frac{{\sum }_{t=1}^{T}{\widehat{u}}_{it}{\widehat{u}}_{jt}}{{\left(\sum_{t=1}^{T}{\widehat{u}}_{it}^{2}\right)}^{1/2}{\left(\sum_{t=1}^{T}{\widehat{u}}_{jt}^{2}\right)}^{1/2}}$$

As in Holly et al. (2010), the CD test is exploited on the residuals of the ADF test of the series.

About testing unit-roots, several panel tests in the presence of cross-dependency have been proposed. The first test (Pesaran, 2007) suggests a cross-sectionally augmented Dickey-Fuller (CADF) test where the standard DF regressions are augmented with cross-sectional averages of lagged levels first differences in the individual series. The author also considers an augmented cross-sectional IPS, CIPS test, representing the sample average of the individual CADF test.

The CIPS test is the following:

$$CIPS\left(N,T\right)=\frac{{\sum }_{i=1}^{N}{t}_{i}\left(N,T\right)}{N}$$

where ${t}_{i}(N,T)$ is the cross-sectionally augmented Dickey-Fuller statistic for the i-th cross-section unit given by the t-ratio of the coefficient of the CADF regression (see Pesaran, 2007). The distribution of the test is not standard, and the critical values are tabulated by the author for different combinations of N and T and are given in Tables II(b) to II(c) in Pesaran (2007).

## Footnotes

1 The ADSL (Asymmetric Digital Subscriber Line) was launched in Italy on the first January 2000 by a private company (Galactica) but on a larger scale on 26 January, when the Italian Telcom TIN (Telecom Italia Network) connected 25 Italian cities.

2 Basically, the private economy, excluding the financial and insurance sectors.

3 It is known, in the literature, that maximum likelihood estimation produces an overestimation due to the endogeneity problem discussed in the previous section. (see Beveren, 2012).

4 See Appendix A2.

5 See Appendix A2.

6 Trentino Alto Adige is splitted in two sub-regions: Bolzano and Trento, according to NUTS 2 classification for Italy.

7 Some regions show a steadily inverse pattern after 2008. For instance, Lazio that is a region highly characterized by services related to the public administration, which is out of our data, and Molise.
